# The ongoing *Streptococcus pyogenes* (Group A *Streptococcus*) outbreak in London, United Kingdom, in December 2022: a molecular epidemiology study

**DOI:** 10.1016/j.cmi.2023.03.001

**Published:** 2023-07

**Authors:** Adela Alcolea-Medina, Luke B. Snell, Christopher Alder, Themoula Charalampous, Tom G.S. Williams, Synnovis Microbiology Laboratory Group, Vasanthini Athitha, Jahaedea Begum, Massimo Bonaiti, Julie Brennan, Lisa Bryan, Albert Cerda, Penelope R. Cliff, Luong Huw Hoang, Tammy V. Merrill, Denitsa Naumova, Rayhan Parvez, Kristine Valle, Sarah White, Diane Wray, Mark K.I. Tan, Noor Al-Yaakoubi, Gul Humayun, William Newsholme, Simon Goldenberg, Gaia Nebbia, Stuart J.D. Neil, Rahul Batra, Jonathan D. Edgeworth

**Affiliations:** 1) Department of Infectious Diseases, King's College London, London, UK; 2) Infection Sciences, Synnovis, London, UK; 3) Centre for Clinical Diagnostics & Infectious Disease Research, Guy's & St. Thomas' NHS Foundation Trust, London, UK; 4) Department of Infection, Guy's & St. Thomas' NHS Foundation Trust, London, UK

**Keywords:** Bacterial typing, Epidemiology, Group A *Streptococcus*, Streptococcus pyogenes, Whole-genome sequencing

## Abstract

**Objectives:**

Epidemiological and whole-genome sequencing analysis of an ongoing outbreak of *Streptococcus pyogenes* (Group A *Streptococcus*) in London (United Kingdom).

**Methods:**

Prospective identification of Group A *Streptococcus* cases from a diagnostic laboratory serving central and south London between 27 November and 10 December 2022. Case notes were reviewed and isolates were retrieved. Case numbers were compared with the previous 5 years. Whole-genome sequencing was performed with long-read, nanopore technology for *emm* typing and identification of superantigen genes. Associations of pathogen-related factors with an invasive disease were assessed by single-variable and multi-variable logistic regression.

**Results:**

Case numbers began increasing in October 2022 from a baseline of 2.0 cases per day, and in December 2022, the average daily case numbers reached 10.8 cases per day, four-fold the number usually seen in winter. A total of 113 cases were identified during the prospective study period. Three quarters (86/113, 76%) were paediatric cases, including 2 deaths. Of 113 cases, 11 (10%) were invasive. In total, 56 isolates were successfully sequenced, including 10 of 11 (91%) invasive isolates. The *emm12* (33/56, 59%) and *emm1* (9/56, 16%) types were predominant, with 7 of 9 (78%) *emm1* isolates being from the M1uk clone. The majority of invasive isolates had superantigen genes *spea* (7/10, 70%) and *spej* (8/10, 80%), whereas, in non-invasive isolates, these superantigen genes were found less frequently (*spea*: 5/46, 11% and *spej:* 7/46, 15%). By multivariable analysis of pathogen-related factors, *spea* (OR 8.9, CI 1.4–57, p 0.020) and *spej* (OR 12, CI 1.8–78, p 0.011) were associated with invasive disease.

**Conclusions:**

*emm12* and *emm1* types predominate in the ongoing outbreak, which mainly affects children. In this outbreak, the *spea* and *spej* superantigen genes are associated with the severity of presentation.

## Introduction

On 2 December 2022, the UK Health Security Agency alerted clinicians to an increase in scarlet fever and invasive disease caused by *Streptococcus pyogenes* (Group A *Streptococcus* [GAS]) [1]. Invasive disease, defined as the isolation of GAS from a normally sterile site [2], has resulted in the death of around 30 children [1]. It is unclear whether this increase is associated with the circulation of particularly invasive types or clones. We, therefore, undertook a review of epidemiology and performed rapid whole-genome sequencing (WGS) of GAS from community and hospital cases in London (United Kingdom).

## Methods

### Study period, population and case details

Cases of GAS were identified and prospectively collected over 2 weeks (27 November until 10 December 2022). Cases were captured from our laboratory reports, which serves 2 adult hospitals, one paediatric hospital and community services for approximately 500,000 people in central and south London. Linked clinical data were retrieved from electronic patient records. Clinical information retrieved included sex, age, area of residence, sampling date, sample site and geographical location of sampling (categorized as general practice, outpatients, emergency department [not admitted] or hospital inpatients). Where the area of residence was not available, the location of the swab was used (*n* = 7). Invasive cases were defined as per Centre for Disease Control definitions [3]. Outcomes included GAS-related hospitalization, ICU admission and death, censored after 23 December 2022. Retrospective reports of GAS isolation for the past 5 years were also retrieved from the laboratory reports. Incidence graphs were made in StataMP (StataCorp LLC, TX). Geospatial data were mapped using Geopandas v0.12.2 (https://geopandas.org/), with cases inside of Greater London (*n* = 101) mapped onto middle layer super output areas (Office of National Statistics). Cases outside Greater London are not shown (*n* = 12).

### WGS of isolates

During the prospective study period, new growths of GAS were identified by scientists in the diagnostic lab and saved for WGS. GAS isolates were then grown on purity plates before being subjected to bead beating for nucleic acid extraction, as previously described [4]. Library preparation and indexing were performed using SQB-RBK110.96 and sequenced on R.9.4.1 flow cells (Oxford Nanopore Technologies). Multi-locus sequence typing was performed using krocus v1 (https://github.com/andrewjpage/krocus). Genome assembly was performed using flye v.2.8.3 (https://github.com/fenderglass/Flye) and polished with medaka v1.7.2 (https://github.com/nanoporetech/medaka). *emm* typing was performed on completed assembly using emmtyper (https://github.com/MDU-PHL/emmtyper) and in parallel by mapping reads with BLAST against the CDC reference dataset (https://www2.cdc.gov/vaccines/biotech/strepblast.asp). Reads were interrogated for the presence of virulence factors using BLAST against Virulence Factor DataBase (http://www.mgc.ac.cn/VFs/), specifically detection of superantigens *smez*, *spej*, *speg*, *spea*, *spec*, *ssa*, *spem*, *spei*, *speh*, *spek* and *spel* genes based on previous reports [5]. Superantigen genes were considered detected if reads mapped with 90% coverage and 90% identity, as previously described for antimicrobial resistance genes [4]. Basecalled and demultiplexed read data are available at the Sequence Read Archive (BioProject: PRJNA914624). The use of samples and linked clinical data was approved by the Research Ethics Committee (18/NW/0584).

### Statistical analysis of the association of pathogen-related factors with invasive disease

Single-variable analysis assessing the association of pathogen-related factors with invasive disease used the likelihood ratio test. In this analysis, age was treated as categorical data: either paediatric (<18 years of age) or adult. *emm* type was categorized into *emm1* or other, due to the previous association of emm1 with invasive disease. Variables with a significance value of p ≤ 0.2 were taken forward to multi-variable analysis, conducted using backward stepwise binary logistic regression. The significance threshold was set at p < 0.05. Analysis was performed using SPSS v28 (IBM, NY).

## Results

### Five-year epidemiological surveillance of laboratory-identified GAS infections

**Fig. 1:**
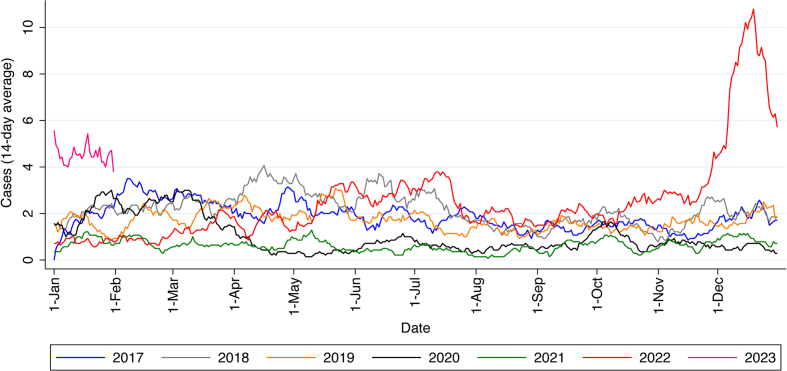
Daily cases (14-day moving average) of laboratory-reported Group A *Streptococcus* isolation from clinical samples between 1 January 2017 and 31 January 2023.

Cases of GAS between 2017 and 2023 are shown in Fig. 1 (see also Tables S1 and S2). GAS infections usually peak in late spring and early summer, as previously described [1]. During the 2 pandemic years (March 2020 to March 2022), GAS cases fell below the monthly nadir seen in previous years. In 2022, an increase in case numbers in December 2022 reached 10.8 cases per day over a four-fold higher average daily case numbers than in December of the pre-pandemic years, amounting to 238 cases in December 2022 in total. Cases still remain higher than the other years as of February 2023.

### Description of GAS cases captured within the study period

A total of 113 cases were identified (Table 1 and Table S3) during the prospective study period of 27 November until 10 December 2022. Of 113 cases, 59 (52%) were female and the median age was 7 years (IQR 4–13), with 86 of 113 (76%) being paediatric cases under 18 years old. Of 113, 11 (10%) were considered invasive. A similar proportion of paediatric cases (7/86, 8%) and adult cases (4/27, 5%) was invasive. No demographic or case details were missing. Geospatial data on incidence are shown in Fig. S1 (see also Table S4).

**Table 1:** Description of cases and summary of typing results

Characteristic	Paediatric		Adult	
*n* (%)	86 (76%)		27 (24%)	
Mean age (y) (IQR)	5 (4–7)		41 (34–57)	
Clinical manifestation				
Non-invasive				
Upper respiratory tract	65 (76%)		9 (33%)	
Skin and soft tissue	12 (14%)		14 (52%)	
Scarlet fever	2 (2%)		0 (0%)	
Invasive				
Lower respiratory tract	5 (6%)		2 (7%)	
Septic arthritis	1 (1%)		1 (4%)	
Necrotizing fasciitis	0 (0%)		1 (4%)	
Bacteraemia	1 (1%)		0 (0%)	
GAS-related outcomes				
Hospitalization	10 (12%)		7 (27%)	
ICU admission	3 (3%)		2 (7%)	
Death	2 (2%)		0 (0%)	
Typing (*n* = 56)	Invasive	Non-invasive	Invasive	Non-invasive
*emm12*	2	24	2	5
*emm1*	5	3	0	1
Other	0	7	1	6

GAS, Group A *Streptococcus*.

Of 113 cases, 17 (15%) were admitted to the hospital, with 2 deaths in children. Most (7/11, 64%) invasive cases were lower respiratory tract infections. Of the 7 cases of invasive respiratory disease, 4 had co-infection with respiratory viruses (human metapneumovirus = 2; influenza = 1, respiratory syncytial virus = 1), 2 did not, and 1 was not tested. Of the 102 non-invasive cases, most (74/102; 73%) were pharyngitis (Table 1).

### WGS analysis and molecular typing

Isolates were retrieved from 65 of 113 (58%) cases. WGS was successful for 56 of 65 (86%) isolates retrieved (Fig. S2), comprising 10 of 11 (91%) invasive cases and 46 of 102 (45%) non-invasive cases. Similar to the whole cohort, the majority of successfully sequenced isolates were from paediatric cases (41/56, 73%), with a median age of 6 years (IQR 4–29), and most (39/56, 70%) were cases of pharyngitis.

Typing data for the 56 sequenced isolates are presented in Table 1 (and Tables S5 and S6). The majority were of 2 *emm* types: *emm12* (33/56, 59%) and *emm1* (9/56, 16%). A higher proportion of invasive disease was caused by emm1 (5/10, 50%), compared with non-invasive disease (4/46, 9%). In addition, the *emm1* type comprised a higher proportion of paediatric (8/41, 20%) compared with adult (1/15, 7%) cases.

The *emm1* types were further characterized to identify whether they were from the M1uk clone, which has previously been associated with increases in scarlet fever and increased superantigen production [6]. Seven of 9 (78%) *emm1* types were highly related to the M1uk clone, sharing 26 of 27 characteristic single nucelotide polymorphisms (SNPs), including 4 of 5 (80%) of the invasive *emm1* isolates. Single SNP differences have also been documented in another study describing the M1uk clone [7]. The remaining 2 of 9 (22%) *emm1* isolates were unrelated to the M1uk clone, lacking any of the M1uk-defining SNPs. GAS isolates from the 2 paediatric deaths were caused by an *emm12* and a non-M1uk *emm1* type.

### Detection of superantigen genes by WGS

Each successfully sequenced isolate was analysed for the presence of 11 superantigen genes (Tables S5 and S7). The superantigen *spea* (streptococcal pyrogenic exotoxin) was found in a majority of invasive isolates (7/10, 70%) but in a minority of non-invasive isolates (5/46, 11%). Similarly, the superantigen *spej* gene was more common in invasive isolates (8/10, 80%), compared with non-invasive isolates (7/46, 15%). Most (7/9, 78%) of *emm1* had *spea* and *spej*, including all (5/5, 100%) of the invasive *emm1* isolates. Most M1uk isolates had *spea* (6/7, 86%) and *spej* (5/7, 71%) detected. Both paediatric deaths carried *spea* and one also carried *spej*.

### Single-variable and multi-variable analysis of pathogen factors associated with invasive disease

From the successfully sequenced isolates, *emm* type, M1uk clone and the presence of 11 superantigen genes were investigated for association with the invasive disease by single-variable analysis (Table 2). The superantigens *spek*, *spel*, *speg* and *spem* were omitted from the analysis due to collinearity. Of pathogen-related factors, *emm1* type (OR 10.5; 95% CI 2.1–52), M1uk clone (OR 9.6l; 95% CI 1.7–52), superantigens *spej* (OR 22; 95% CI 3.9–27) and *spea* (OR 19; 95% CI 3.7–99) were significantly associated with invasive disease (p < 0.05).

**Table 2:** Single-variable and multi-variable logistic regression analysis of the association of pathogen-related factors with invasive disease

Variable	OR (95% CI)	P
Single-variable analysis		
*emm1* type	11 (2.1–52)	0.004
M1Uk clone	9.6 (1.7–54)	0.010
Superantigen genes		
*smez*	0.20 (0.011–3.5)	0.27
*spej*	22 (3.9–127)	<0.001
*spea*	19 (3.7–99)	<0.001
*spec*	0.28 (0.40–1.9)	0.20
*ssa*	0.26 (0.064–1.1)	0.065
spei	0.92 (0.21–4.1)	0.91
speh	1.9 (0.37–10)	0.44
Multi-variable analysis: final model		
spea	8.9 (1.4–57)	0.020
spej	12 (1.8–78)	0.011

For the binary logistic regression model, pathogen-related factors from the univariable analysis with a p value ≤ 0.2 were taken forward for backwards step selection. Variables in the multi-variable model therefore included *emm* type, M1uk clone and genes for superantigens *spej*, *spea*, *spec* and *ssa*. Table 2 shows the final model with 2 variables retaining significance for association with invasive disease: *spea* (OR 8.9; 95% CI 1.4–57; p = 0.020) and *spej* (OR 12; 95% CI 1.8–78; p = 0.011). The final model can predict invasiveness 89% correctly (96% of non-invasive and 60% of invasive cases).

## Discussion

The epidemiology presented here from London reflects the pattern seen nationally with a dramatic increase in GAS infections in late 2022, after 2 years of below-average incidence during the COVID-19 pandemic. The reasons for this epidemiological pattern are unclear, but postulated to be caused by decreased GAS exposure, particularly amongst children due to non-pharmaceutical interventions (social distancing and/or masking) during the COVID-19 pandemic, leading to lower levels of immunity [1]. This may also be compounded by high circulating rates of respiratory viruses this winter, including influenza and COVID-19, which may pre-dispose to subsequent GAS infections [8,9]. Although first recognized in the UK, an increase in GAS infections is now being reported in other countries in the European region (Ireland, France, the Netherlands and Finland) [10] and in the United States [11].

There is an association between certain *emm* types, high superantigen production and invasive disease [12]. The majority (75%) of isolates sequenced were *emm1* and *emm12* types, similar to what has been reported nationally [1]. *Emm1* types were overrepresented in the invasive cases, and the majority of the *emm1* isolates were of the M1uk clone. Before the pandemic, M1uk was recognized to comprise the majority of UK emm1 isolates, with high superantigen production [6]. This clone has also been reported in North America [7].

To disentangle the association of pathogen factors with invasive disease, multi-variable analysis was conducted, showing that the association is attributable to *spea* and *spej* superantigen rather than the other analysed pathogen-related factors. *spea* and *spej* superantigen production may therefore explain the pathological mechanisms behind the invasive disease in this outbreak. Alternatively, other unassessed pathogen factors that cosegregate with these genes may play a mechanistic role.

As sequence typing data were obtained only for half the isolates, our results may not be representative of the outbreak nationally. In addition, viral respiratory infections were not systematically assessed, leaving us unable to comment on any association with GAS. Larger studies are required to discriminate the overlapping associations between invasive diseases, types, clones and superantigen carriage. Replicating these findings in other regions will be important to confirm whether increased incidence is related to these pathogen-specific factors, such as superantigens and hypervirulent clones.

This study illustrates the benefit of embedding pathogen sequencing in diagnostic laboratories, allowing rapid outbreak investigation to inform clinical and public health teams. Meanwhile, clinicians in affected areas are recommended to consider GAS for presentations usually due to respiratory viral infections [1].

## Transparency declaration

The work was funded by the 10.13039/501100000265Medical Research Council (LBS: MR/W025140/1, GN: MR/T005416/1, JDE, AA-M, RB, 10.13039/100015876GN, 10.13039/501100004145LBS, TC: MC_PC_19,041). JDE has received funding from the Guy's and St. Thomas' Charity (TR130505). AA-M and members of the Synnovis Microbiology Laboratory Group are full-time employees of Synnovis. JDE holds a part-time employment contract with Oxford Nanopore Technologies that commenced in October 2022. Guy's & St Thomas' NHS Foundation Trust signed a commercial collaboration agreement with Oxford Nanopore Technology in September 2022. WN, CA, GH, MT, NA-Y, SDJN, SG and TGSW have no conflicts of interest to declare.

## Author contributions

AA-M, LBS, CA, TC and TGSW were involved in conceptualization, data acquisition, methodology and formal analysis. AA-M and LBS were involved in writing—original draft. The Synnovis Microbiology Laboratory Group, MT, GH and NA-Y were involved in the investigation. WN, SDJN, SG, GN and JDE were involved in supervision and writing—review and editing. RB was involved in project administration and resources. AA-M, 10.13039/501100004145LBS, 10.13039/100006922TC, SDJN, 10.13039/100015876GN, RB and JDE were involved in funding acquisition.

## Appendix A

### Supplementary data

Synnovis Microbiology Laboratory Group, list of contributing authors in alphabetical order by surname:

Vasanthini Athitha, Jahadea Begum, Massimo Bonaiti, Julie Brennan, Lisa Bryan, Albert Cerda, Penelope R. Cliff, Luong Huw Hoang, Tammy V. Merrill, Denitsa Naumova, Rayhan Parvez, Kristine Valle, Sarah White, Diane Wray.
